# Is puberty a risk factor for back pain in the young? a systematic critical literature review

**DOI:** 10.1186/s12998-014-0027-6

**Published:** 2014-10-15

**Authors:** Arnaud Lardon, Charlotte Leboeuf-Yde, Christine Le Scanff, Niels Wedderkopp

**Affiliations:** 1 EA 4532 CIAMS, Université Paris-Sud, UFR STAPS, Orsay, 91405 France; 2 Institut Franco-Européen de Chiropraxie, 24 Bld Paul Vaillant Couturier, 94200 Ivry sur Seine, France; 3 Research Department Spinecenter of Southern Denmark Hospital, Lillebélt Middelfart, Denmark; 4 Sport medicine clinic, orthopedic dep., Hospital of Lillebaelt, Institute of Regional Health, Service Research and Center for Research in Childhood Health, University of Southern Denmark, Odense, Denmark

**Keywords:** Back pain, Puberty, Adolescent, Cause, Aetiology, Systematic review

## Abstract

**Background:**

Back pain is a common condition that starts early in life and seems to increase markedly during puberty. A systematic review was performed in order to investigate the link between puberty and back pain, using some Bradford Hill criteria for causality.

**Objectives:**

We sought to obtain answers to the following questions: 1) Is there an association between puberty and back pain? If so, how strong is this association? And do the results remain unchanged also when controlling for age and sex? 2) Are the results of the studies consistent? 3) Is there a dose-response, showing a link between the increasing stages of puberty and the subsequent prevalence of back pain? 4) Is there a temporal link between puberty and back pain?

**Design:**

A systematic critical literature review.

**Methods:**

Systematic searches were made in March 2014 in PubMed, Embase, CINAHL and PsycINFO including longitudinal or cross-sectional studies on back pain for subjects <19 years, written in French or English. The review process followed the AMSTAR recommendations. Interpretation was made using some of the Bradford-Hill criteria for causality.

**Results:**

Four articles reporting five studies were included, two of which were longitudinal. 1) Some studies show a weak and others a strong positive association between puberty and back pain, which remains after controlling for age and sex; 2) Results were consistent across the studies; 3) There was a linear increase of back pain according to the stage of puberty 4) Temporality has not been sufficiently studied.

**Conclusion:**

All our criteria for causality were fulfilled or somewhat fulfilled indicating the possibility of a causal link between puberty and back pain. Future research should focus on specific hypotheses, for example investigating if there could be a hormonal or a biomechanical aspect to the development of back pain at this time of life.

**Electronic supplementary material:**

The online version of this article (doi:10.1186/s12998-014-0027-6) contains supplementary material, which is available to authorized users.

## Background

### Back pain in young age

It has previously been established that back pain starts during childhood [1]-[4]. According to two recent systematic literature reviews [1],[2], the lifetime prevalence increases between the ages of 7 and 12 (on average from 1% to 17%) to reach the adult level around the age of 20 [5]. In relation to low back pain, it appears that puberty is the time for a rapid increase. Girls start puberty earlier than boys, which may explain why they report back pain earlier than boys [5].

### Puberty and back pain

The time of puberty is the transition period from childhood to adulthood and over only a few years, both body and soul will undergo many changes. The most apparent morphological differences are increased height and a change in body composition. It has been proposed that these may impact back pain [6]-[8]. The growth spurt, defined as an average gain of 10-cm per year [9], could be considered a particularly vulnerable period due to sudden mechanical loading changes on the spine. According to a longitudinal study [10], in a healthy population of white girls, the fat mass was shown to increase at the end stage of pubertal development. If this fat replaces active muscle fibers, this too could result in back problems.

Puberty is also an important period for hormonal development and it has been shown that the different pubertal stages are positively associated with the level of sex hormones in both boys and girls [11]. These hormones play a role in pain perception, as demonstrated in adults [12]-[15]. It has been shown, for example, that hormone replacement therapy is linked to back pain in postmenopausal women [13] and that the perception of experimentally induced pain varies throughout the menstrual cycle [15]. It is also known that pain perception is different for men and women of reproductive age [14], but no such difference was found in elderly surgical patients [12]. As a consequence, it has also been suggested that hormonal changes appearing at puberty may influence the perception of pain. However, we were unable to find any research published in English on this topic.

There are, therefore, several potential reasons why back problems could develop or, at least, become more readily felt during the period of puberty than in earlier childhood. For this reason, a systematic critical literature review was undertaken in order to investigate whether there is any evidence in relation to puberty as a cause of back pain.

### Research questions

Specifically, we sought to obtain answers to the following questions:

1. Is there an association between puberty and back pain? If so, how strong is this association? Will such an association withstand the control of age and sex?
2. Are results of the studies consistent?
3. Is there a dose-response, showing a positive link between the increasing stages of puberty and the prevalence of back pain?
4. Is there a temporal link between puberty and back pain?

## Methods

The authors performed a systematic critical literature review to identify, evaluate and summarize the evidence on whether puberty could be a cause of back pain in the young.

### Search

Searches were made in PubMed, PsycINFO, CINAHL and Embase databases for articles published until March 2014 without limits in relation to language or time. The search was uncomplicated; we used the following search terms as free text and MeSH terms: -puberty-, pubert*- and -back pain-. To select appropriate search terms, the first author consulted a documentalist at the University of Paris-Sud.

### Eligibility criteria

Inclusion criteria related to type of study, study subjects, and target condition. Specifically, longitudinal and cross sectional studies were accepted if they were written in English or French. We included studies of children and/or adolescents below the age of 19, in order to capture both the pre-pubertal and pubertal periods. The target condition was back pain that should be defined independently and not included in a generic term, such as a musculoskeletal pain.

### Screening

**Figure 1:**
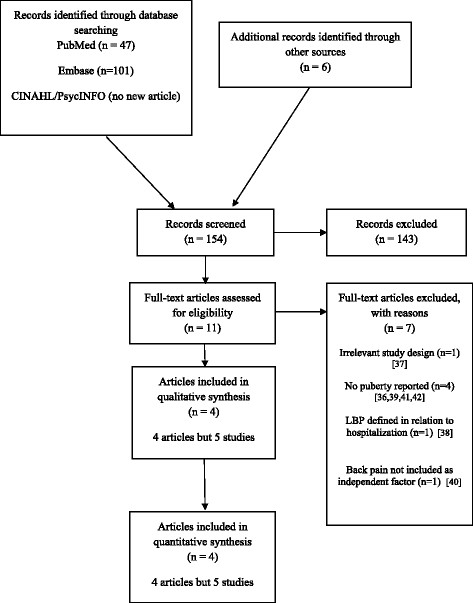
**PRISMA 2009 Flow Diagram.**

The first author read the titles and abstracts to select the relevant full texts. He did an additional search, tracking references from articles and also eliminated any duplicates. The relevant full texts were thereafter read independently by the first and second authors to verify if they could be included in the review. The flow of the study has been reported according to the PRISMA 2009 flow diagram (Figure-1). Excluded articles and the reasons for exclusion have been listed in this figure.

### Methodological quality assessment

A specific checklist for this topic was developed in relation to the needs of this study. This checklist was divided into three parts (description, quality and results). The user-friendliness and relevance of the first two grids were tested independently by the authors, after which improvements were made and then tested again until found suitable. In addition, we followed the criteria for systematic reviews listed in AMSTAR [16], with the exception that we did not take into account 'conflict of interest', as there would be no financial gain from the results of this type of study.

The descriptive items are listed in Table-1. As an already existing quality checklist that fulfilled our needs could not be found, one had to be designed (Table-2). This resulted in six main topics relating to methodological quality and bias, resembling the checklist of Landrivon [17], which is an adaptation of the -Critical Appraisal Worksheet- from the Center for Clinical Epidemiology and Biostatistics of Newcastle University. An additional item (reliability) was taken from the SIGN checklist [18]. The quality assessment was relevant for the purposes of our study but would not necessarily describe the quality in relation to the authors- original research question(s), which may have been different to our research questions. The rationale for this checklist is explained below:

1. Study sample. We wanted to know if the sample appeared to be representative of the corresponding target population, to determine if the results of these studies could be generalized. If the response rate was inferior to 80%, we expected that authors would have investigated potential response bias to see if the absence of non-responders was likely to affect the outcome. We calculated the response rates in the subsequent surveys on the basis of the invited number of study subjects at baseline and not on the basis of the number of participants in the preceding survey. The latter method is often used in studies but does not provide transparent information on the real proportion of participants of the study sample.
2. Data collection. In order to limit expectation bias, two different persons, blind to each other-s findings, should have collected information on back pain and puberty, unless at least one of these items was collected in a questionnaire.
3. The studied factor: puberty. The concept of puberty should be clearly defined, i.e. explaining how the puberty score was obtained and using a measure, stated to be valid and reliable.
4. The outcome measure: back pain. The outcome measure should be clearly defined. It should be explained how back pain was assessed and the recall period should be specified. In order to limit memory decay, the recall period should be minimized [3]. It was decided to limit this period to one month because it has been shown previously in adults that there is a good correlation between data obtained in adults through real-time data capture by text-messages and a retrospective telephone interview with a one-month recall period [19]. We considered that children would have fairly short recall ability but hoped that one-month would be acceptable also for them. Since there is no gold standard for -back pain-, validity could not be established. At baseline, it should also be specified if study subjects were pain free and preferably, if they had never previously had back pain, in order to establish temporality.
5. Potential modifiers/confounders. We also investigated if associations between puberty and back pain were controlled for possible confounders or modifiers, particularly age and sex. These are the only ones known to be linked with back pain in children, as back pain accelerates at this time in life and girls are more likely to report back pain than boys.
6. Finally, in the cases where the biological gradient (dose-response) was investigated, it was assessed if it had been submitted to a statistical test for trend and if the assumption for making such a test had been met.

**Table 1:** **Description of four articles reporting on five studies on puberty and back pain**

Reference number	[33]	[33]	[35]	[34]	[32]
1^st^ author	Jansens	Jansens	Wedderkopp	LeResche	Hulsegge
Year	2011	2011	2005	2005	2011
Country	Netherland	USA	Denmark	USA	Netherland
Design	Longitudinal	Longitudinal	Cross-sectional	Cross-sectional	Cross-sectional
Sample size	BL: 2935	BL: 4079	419	3101	2698
	FU: 1816	FU: 1817			
Response rate	BL:76%	BL: 49%	51%	49%	66%
	1^st^ FU: 73%	FU: 45%			
	2^nd^ FU: 62%				
Age	BL: 11.1	BL: 11	8-10 and 14-16	11-17	11
	FU: 15.1	FU: 14			
Sex distribution (% of girls)	51	51	100	50	49
Description of puberty	4 stages	4 stages	5 stages	4 stages	4 stages
Data collection for puberty	Questionnaire PDS	Telephone itw PDS	Physical examination Tanner stages	Telephone itw PDS	Questionnaire PDS
Description of back pain	BP = frequency	BP = frequency	BP = areas (LBP/MBP/NP)	BP = duration (>one day or more)	BP = duration (>1-month)
Data collection for back pain	Questionnaire	Telephone itw	Semi-structured itw	Telephone itw	Questionnaire
Recall period for back	3-months	3-months	1-month	3-months	12-months
Other extrinsic factors than age and sex	Pubertal status at BL	Pubertal status at BL	Overweight Smoke	Previous pain Depression	Not in the analysis for back pain
	BP at BL	BP at BL			
Dose-response analysis possible	Prevalence estimate AND frequency of symptoms in relation to puberty stages	Prevalence estimate AND frequency of symptoms in relation to puberty stages	Prevalence estimate in relation to puberty stages	Prevalence estimate in relation to puberty stages	Prevalence estimate in relation to puberty stages

BL = baseline, FU = follow up, Itw = interview, PDS = pubertal development scales, BP = back pain.

**Table 2:** **Quality assessment of four articles reporting on five studies on puberty and back pain**

		Jansens[33](Netherlands)	Jansens[33](USA)	Wedderkopp[35]	LeResche[34]	Hulsegge[32]
1. Study sample	- Was the sampling likely to be suitable to obtain a group representative of the corresponding general population?	Yes (Probably)	Yes (Probably)	Yes (Probably)	Yes (Probably)	Yes (Probably)
	- If participation at BL or at FU < 80%, was response bias investigated?	Yes	Yes	Yes	Yes	No
	- Inclusion criteria, were age groups appropriate to study the whole period of puberty?	Yes	Yes	Yes	Yes	Not really
2. Data collec-tion	- Data for BP and puberty were collected independently by 2 different persons or by at least 1 questionnaire?	Yes	No	Yes	No	Yes
3. Puberty	- Clearly defined?	Yes	Yes	Yes	Yes	Yes
	- Description of how puberty stage was determined?	Yes	Yes	Yes	Yes	Yes
	-Reference provided for validity of test?	No	No	No	Yes	Yes
	-Reference provided for reliability of test?	Yes	Yes	No	No	No
4. Back pain	- Clearly defined?	Yes	Yes	Yes	Yes	Yes
	- Description of how BP was assessed?	Yes	Yes	Yes	Yes	Yes
	- Recall period (<= 1-month)?	No	No	Yes	No	No
	- Was BP at BL taken into account?	Yes	Yes	NA	NA	NA
5. Control for age and sex	- Was the association puberty- BP controlled for age?	Yes	Yes	Stratified analysis	Yes	Yes
	- for sex?	Yes	Yes	NA	Stratified analysis	Yes
6. Stat. analysis	- Was dose-response prevalence estimated in relation to puberty stages investigated?	Yes	Yes	Yes	Yes	No
	- Was dose-response of frequency or severity in relation to puberty stages investigated?	No	No	NA	NA	NA
	- Was dose-response tested for trend?	Yes	Yes	Yes	Yes	Yes
Score (0-17)%		14/17	13/17	12/14	12/15	10/15
		82%	76%	86%	80%	67%

(listed by the quality score).

BL = baseline, FU = follow up, BP = back pain, NA = not applicable.

A summary of the results is shown in Table-3. These items related to various aspects of causality, using the method for causal assessment developed by Roffey *et al.*[20]-[24] and Wai *et al.*[25]-[27], which in turn was based on relevant criteria for causality previously described by Bradford Hill [28]. As in their reviews, the following elements were considered: strength of the association, dose response, and temporality (further described below). -Plausibility- was not taken into consideration, as this was evident, and also not -experiment-, as this was irrelevant in observational studies, but -consistency- was added. Dose-response was taken into account both in cross-sectional and longitudinal studies but temporality could only be investigated in longitudinal studies, provided that the back pain variable had been defined clearly in terms of prior absence of pain.

**Table 3:** **Summary of results from four articles reporting on five studies on puberty and back pain**

	Jansens[33]	Jansens[33]	Wedderkopp	LeResche			Hulsegge
	Netherland	USA	[35]	[34]			[32]
**Prevalence estimates of back pain**							
(Puberty stage: Number or percentage of children with BP/Number of individuals)			Stage 1 : 52/179				
	? : 15%/992 girls	? : 30.5%/1069 girls	Stage 2 : 22/68	Puberty stage <2 : ?/155			? : 3.2%/1313 girls
			Stage3 : 8/16	Puberty stage 2 to 3 : ?/?			
	? : 11.1%/1004 boys	? : 22.1%/1018 boys	Stage 4 : 40/76	Puberty stage > 3: ?/594			? : 2.1%/1325 boys
			Stage 5 : 38/74				
**OR obtained by logistic regression** (Dependent variable : back pain/independent variable: puberty stage)	Unadjusted OR = 1.34 * (95% CI 1.13-1.57)	Unadjusted OR = 1.61* (95% CI 1.30-1.99)	Unadjusted values: Not provided	Boys: Unadjusted OR = 1.9* (p < 0.0001)			Unadjusted values: Not provided
	Adjusted OR = 1.24 * (95% CI 1.04-1.46) ^a^	Adjusted OR = 1.52* (95% CI 1.22-1.89)^a^	Adjusted OR = 1.2* (95% CI 1.2-1.4)^b^	Girls: Unadjusted OR OR = 2.0* (p < 0.0001)			Both: Adjusted OR = 1.23 (95% CI 0.77-1.96) ^c^
				Adjusted values not provided, however association said to have remained when controlling on age and parental education			Boys: Adjusted OR = 2.86 * (95% CI 1.14-7.15) ^c^
							Girls: Adjusted OR = 1.09 (95% CI 0.65-1.84)^c^
**Assumption of linearity fulfilled for logistic regression**	Yes	Yes	Yes	Yes			Not reported
	*For both sexes:*	*For both sexes:*	*For girls only:*	*For girls and boys:*			*For boys only:*
	Unadjusted values:	Unadjusted values:	Unadjusted values:	Unadjusted values:			Unadjusted values :
**Dose-response** (Odds ratios by puberty stage)	Puberty stage 1 (Index)	Puberty stage 1 (Index)	Puberty stage 1 (Index)				
	Puberty stage 2 OR = 1.34 ^#^	Puberty stage 2 OR = 1.61^#^	Puberty stage 2 OR = 1.1 (95%CI0.6-2.0)	Puberty stage	girls	boys	
	Puberty stage 3 OR = 1.79 ^#^	Puberty stage 3 OR = 2.59^#^	Puberty stage 3 OR = 2.3 (95%CI0.8-6.5)	1	(Index)	(index)	not provided
	Puberty stage 4 OR = 2.4^#^	Puberty stage 4 OR = 4.17^#^	Puberty stage 4 OR = 2.6 (95%CI1.5-4.7)	2	OR = 2^#^	OR = 1.9^#^	
	Adjusted values^a^:	Adjusted values^a^:	Puberty stage 5 OR = 2.4 (95%CI1.4-4.3)	3	OR = 4^#^	OR = 3.61^#^	
				4	OR = 7.99^#^	OR = 6.86^#^	
	Puberty stage 1 (Index)	Puberty stage 1 (Index)	Adjusted values^b^:	Adjusted values:			Adjusted values^c^
	Puberty stage 2 OR = 1.24 ^#^	Puberty stage 2 OR = 1.52 ^#^	Puberty stage 1 (Index)	not provided			Puberty stage 1 (Index)
	Puberty stage 3 OR = 1.54 ^#^	Puberty stage 3 OR = 2.3 ^#^	Puberty stage 2 OR = 1.1 (95%CI 0.7-2.0)				Puberty Stage 2 OR = 2.86^#^
	Puberty stage 4 OR = 1.9 ^#^	Puberty stage 4 OR = 3.5 ^#^	Puberty stage 3 OR = 1.6 (95%CI 0.5-4.6)				Puberty Stage 3 OR = 8.18^#^
			Puberty stage 4 OR = 2.0 (95%CI 1.3-3.5)*				Puberty Stage 4 OR = 23.4^#^
			Puberty stage 5 OR = 2.1 (95%CI 1.1-4.1)*				
**Strength of association based on index versus last stage of puberty**	Puberty stage 4	Puberty stage 4	Puberty stage 5	Puberty stage 4			Puberty stage 4
	Unadjusted OR = 2.4^#^	Unadjusted OR = 4.17^#^	Unadjusted OR = 2.4 (95%CI 1.4-4.3)	Unadjusted OR = 7.99^#^ for girls			
	Adjusted OR = 1.9 ^# a^	Adjusted OR = 3.5 ^#^	Adjusted OR = 2.1 (95%CI 1.1-4.1)* ^b^	Unadjusted OR = 6.86^#^ for boys			Adjusted OR = 23.4^# c^
**Temporality tested**	Positive association noted between puberty stage at baseline and BP at follow-up after adjustment for back pain at baseline	Positive association noted between puberty stage at baseline and BP at follow-up after adjustment for back pain at baseline	NA	NA			NA

OR = Odds ratio.

BP = Back pain.

CI = confidence interval.

* statistically significant.

^#^ estimations of odds ratio (comparison of each stages compared to the index value) calculated from logistic regression provided in article.

& ORs not calculated for girls and both sexes together because not significant.

(a) adjusted for sex and back pain.

(b) adjusted for overweight and smoking/stratified by age.

(c) adjusted for age and sex.

Each article that fulfilled the inclusion criteria was independently reviewed by two authors blind to each other-s findings using the checklist. For each item, the authors specified if the checklist item was present, absent or not applicable. If the two reviewers disagreed, the third author would be consulted. One of the articles was not reviewed by the second author, who was a co-author of that study. In addition, the statistical aspect was reviewed by the last author.

### Analysis

The results in the grid were summarized and a quality percentage score was calculated for the second part of the checklist. The studies were classified, first according to their design, and then by their methodological quality scores. They were thereafter scrutinized in relation to the research objectives. No cut-point for minimum standard of quality was established. Instead, we intended to rely more on the results of the better studies than those of lower scores, if studies with the lowest scores reported findings that deviated from the better studies [29].

The result table included a descriptive item relating to the prevalence of back pain in relation to the various subgroups that had been analyzed (sex, puberty stages). It also included the unadjusted and adjusted odds ratio (ORs) obtained in the logistic regression.

Causality was explored on the basis of the following Bradford Hill criteria [28].

1. Strength of association. Estimates of associations were scrutinized in the text and tables, using the ORs of the latest puberty stage compared to the index value. When only the logistic regression estimate had been provided, we used it to estimate the values for each pubertal stage based on the initial estimate (see Additional file 1 for this procedure). This method did not make it possible to provide 95% confidence interval (CI) if authors did not include information needed for this in their articles. However, when interpreting the results, values indicating the strength of the association can only be trusted if the assumption for linearity between the logit of the probability of -positive- response in the dependent variable and the continuous independent variable (in this case puberty stages treated as a continuous variable to test for trend) have not been obviously violated. We therefore included also a checklist item for this and verified if this had been reported in the text. ORs were considered to be significantly positive if 1 was not included in the confidence interval or if authors defined significance through a p-value. ORs between 1.0 and 2.4 were considered to be weak, whereas ORs between 2.5 and 3.9 were considered to be moderate and strong if they were larger than 4.0 [30]. In addition, we investigated if the authors had controlled for, at least, age and sex in their analyses. If other variables had been included in the analyses, these were also noted. This item was relevant both in cross-sectional and longitudinal studies.
2. Consistency of findings. Consistency was arbitrarily deemed to be strong if 75% of studies were in agreement, and if at least two such studies were of high quality [31]. This item was relevant both in cross-sectional and longitudinal studies.
3. Dose-response. We studied the dose-response between the various puberty stages and the prevalence of back pain. The presence of a dose-response link could also be indicated if puberty stages and the severity or frequency of back pain showed a gradual positive association. Such a positive link was acceptable if a clear step-wise increase of estimates was visualized, in particular if this incline was shown to be statistically significant. Statistical significance could be shown by odds ratio of separate stages of puberty not being inside the confidence intervals of some of the other stages, or by some type of test for trend. This item was relevant both in cross sectional and longitudinal studies. These results were dependent either on data provided by the authors or on our estimations of the different stages.
4. Temporality. In longitudinal studies, it would be necessary to include children who had never previously had back pain at baseline in order to see if the onset of puberty would be associated with the development of back pain. As back pain is relatively rare in childhood and lifetime data on back pain would be difficult to collect at this age, we considered it acceptable also to have defined children as having or not having back pain at the time around baseline, as probably the majority without back pain at that time would have been truly back pain free also prior to the study.

We did not perform a meta-analysis because the definitions and reporting of back pain in the articles were found to be heterogeneous. Instead, findings were summarized in a narrative fashion. The final conclusion on causality was obtained in a subjective manner on the basis of the weight of all evidence in relation to strength of association, consistency, dose-response, and temporality.

## Results

### Description of studies - general

A total of 154 potentially relevant articles were identified, 47 in PubMed, 101 in Embase and six by citation tracking. Eleven full texts were assessed for eligibility, resulting in four articles reporting on five studies suitable for review [32]-[35] whereas seven articles [36]-[42] were excluded for various reasons (Figure-1).

### Brief description of articles

As seen in Table-1, these articles were published between 2005 and 2011. Two studies were from North America and the remaining three from Europe. The ages of participants ranged from 11 to 17-years. Three studies were cross-sectional in type and one article reported on two longitudinal studies, one conducted in Europe and the other in USA. These two studies started with children aged 11 who were surveyed again at least three years later. The sample sizes in the five studies ranged from circa 400 to approximately 4000, and the calculated response rates ranged between 45% and 66%. Two of the studies dealt with puberty and back pain as their major research topic. The others included back pain among other definitions of pain. Four studies included both boys and girls and one included only girls.

The first included article, by Hulsegge *et al.*[32], is also a cross sectional study in which 2698 children (response rate 66%) aged 11 were included. The aim was to study if various potential risk factors (one of which was puberty) were associated with musculoskeletal complaints, including back pain.

The second article, by Jansens *et al.*[33], consists of two longitudinal studies, one performed in the Netherlands in which 2935 children were included (response rate 76% at baseline and 62% at follow-up) and the other in the USA consisting of 4079 children (response rate 49% at baseline and 45% at follow-up). Boys and girls were reviewed three times from the ages of 11 to 15 (Netherlands) and twice from 11 to 14 (USA). The work was based on the hypothesis that pubertal maturation at baseline is a risk factor for later development of back pain.

The third article, by LeResche *et al.*[34], is a cross-sectional study that included 3101 boys and girls aged from 11 to 17-years old (response rate 49%). The relationship between back pain and pubertal development was assessed.

The last article, by Wedderkopp *et al.*[35], is a cross sectional study that included 254 girls aged 8-10 years and 165 girls aged 14-16 years (response rate 51%). The authors assessed the link between back pain and the different pubertal stages.

Details about the definition of puberty, definition of back pain, extrinsic factors, dose-response and temporality are reported below in the description of studies section.

### Description of studies

#### Definition of puberty

Two classification methods of the various pubertal stages were used: the Pubertal Development Scale (PDS) and the Tanner classification. The first one divides puberty into 4 stages (no development = 1, development barely begun = 2, development definitively underway = 3 and development already completed = 4). To determine the relevant stage, five characteristics for each sex have to be assessed (growth spurt, skin changes and body hair for boys and girls, breast development and menarche in girls, voice change and facial hair growth in boys). The second method, a modified Tanner scale, divides puberty into 5 stages based on the breast development (stage 1 = not started puberty, stage 2 = just starting, stages 3 and 4 = in pubertal development, stage 5 = puberty ended). One study used the Tanner scale [35] whereas the four others used the PDS [32]-[34]. The data for puberty were collected by physical examination [35], telephone interviews [33],[34] or by questionnaires [32],[33] (Table-1).

#### Definition of back pain

The definition of back pain was not homogeneous between the articles. Wedderkopp *et al*. [35] used areas of discomfort in the back (low back, middle back or neck pain), the two longitudinal studies [33] took into account the frequency of back pain, whereas LeResche *et al.*[34] and Hulsegge *et al*. [32] focused on duration of back pain. Hulsegge *et al*. [32] were the only authors to define back pain as long lasting i.e. a duration superior to 1-month in the past year. This information on back pain was collected using interview [35], telephone interviews [33],[34] or questionnaires [32],[33]. In the studies, the recall period for back pain varied from one month to twelve months.

#### Extrinsic factors included in the analysis

All authors controlled their results in relation to age and sex (when relevant). In addition, other extrinsic factors that were used for control were: pubertal status at baseline [33], previous pain [33],[34], depression [34], overweight [35] and smoking [35] (Table-1).

#### Dose-response

All the studies performed a test for dose-response using logistic regression where the association between puberty stages and back pain was considered to be (at least) relatively linear.

#### Temporality

Temporality could be somewhat explored in the two longitudinal studies because the authors controlled for pubertal status at baseline. They included, at baseline, children with or without back pain. However, they did not clearly identify children who, at baseline, had never previously experienced back pain, which would have been necessary to identify true incidence of back pain.

### Methodological quality for the needs of this review

The quality scores were between 67% and 86% (Table-2).

#### Studied factor: puberty

All the authors clearly defined puberty and how a puberty stage was determined, using either the Tanner scale or the PDS which has been shown to have a reasonably good correlation with physician ratings of Tanner Scale stages [43], the latter being considered as the gold standard.

#### Outcome measure: back pain

The back pain variables were always clearly defined and the method of data collection explained. Memory decay could have been an issue in four studies as they used a recall period superior to one month. The two longitudinal studies [33] did not have a definition of absolute absence of back pain at baseline but took presence/absence of back pain at baseline into account in their analyses.

#### Data collection

Data on back pain and puberty were clearly collected independently of each other in three of the studies [32],[33],[35]. In the two remaining [33],[34], it is not known if answers could have been biased during data collection, because all information was obtained by telephone interview and it is not known how this risk was dealt with during data collection.

#### Study sample

The sampling methods were considered adequate in all studies and only one failed to investigate the possibility of response bias. In all studies but one, the age groups seemed relevant (Table-2). The exception being one study [32], in which a large proportion would probably have been pre or early pubertal making it difficult to compare children in different stages of development.

#### Potential modifiers

When relevant, age and sex were included in the analysis, either in a multivariate analysis or through stratification.

### Study results

#### Strength of the association

As can be seen in Table-3, the five studies included in this review all reported positive associations between puberty and back pain. The estimated unadjusted values ranged between 2.4 [33] and 7.99 [34]; both estimated ORs refer to the difference between the index and the final puberty stage (calculated from the OR obtained by logistic regression). No obvious differences were generally noted between the unadjusted and adjusted values. A particularly high OR was reported by Wedderkopp *et al.*[35] for the unadjusted OR of 14.6 (95% CI 3.8-56.6), referring to a specific subgroup of back pain, namely to low back pain.

#### Consistency

All studies reported statistically significant positive associations, although in one of the studies, this association was found only in boys and not in girls. All other results of the studies are in agreement and showed that puberty and back pain are associated.

#### Dose-response

All the five studies, reported on dose-response and all noted a positive gradient of back pain reporting in relation to increasing level of puberty. Again, in one of the studies [32] this was not the case for the girls.

In all studies the estimates indicated a gradual increase of back pain by puberty stage, although estimates were generally not surrounded by 95% CI. In one study [35], CIs were shown but there was overlap between these for all stages. Nevertheless, a test for trend using logistic regression did however confirm linearity.

#### Temporality

In relation to temporality, which could have been dealt with in the two longitudinal studies, none of the studies had separately identified children who were truly back pain free at baseline, but presence/absence of back pain at baseline was taken into account in the analyses of the two longitudinal studies. The results indicate that puberty was associated with the onset of back pain, but whether this was a first time onset or a recurring event is not known.

## Discussion

### Summary of findings

To our knowledge, this is the first literature review on the association between puberty and back pain. In relation to the Bradford Hill criteria, we found two weak associations and three that were strong (ranged 2.4 to 7.99), consistency of findings, a positive biological gradient (dose-response), and some evidence of temporality. These results indicate a clear association between puberty and back pain and that there are several indications towards a causal links. Further, when the researchers controlled for age, the results did not change, indicating that the link between puberty and back pain is not merely the result of the accumulation of other (unknown) risk factors that would appear with age. Nevertheless, there would be many potential risk factors that could be candidates for future research, particularly if they are linked with age. Moreover, it was noted that sex, mostly, did not have an influence on the results.

To establish causality, dose-response is an important criterion. When using logistic regression, an OR is reported from which it is possible to extrapolate the subsequent values. However, this method only provides correct results if the assumption for linearity has been met. This is the reason why we could not take into account the result from one of the studies on this issue (Table-3). Furthermore, to study dose-response it was necessary to have access to children at different stages of puberty and to establish if their prevalence of back pain increased with the subsequent stages. Information on frequency and severity of back pain was collected in the two longitudinal studies but not further used in the analyses, meaning that some possibilities to study worsening of back pain with increased puberty were lost.

These results indicate that puberty may play an etiological role in the development of at least some back pain. These findings, however, do not bring any new information on what aspects of puberty, specifically, may contribute to back pain. Several possibilities spring to mind such as growth spurt, altered pain perception, psychological changes, or an altered lifestyle, which could all contribute, independently or together. Also, as the association is not strong, there would be other competing factors that can explain the onset of back pain.

### Methodological considerations

Five studies were found, all with high or relatively high quality scores. However, the study with the somewhat lower score, contrary to the others, did not find a clear positive association between puberty and back pain for both sexes, but only for boys [32]. This study included children who were all aged 11, which meant that there might not have been a wide enough age range to cover all stages of puberty. It also defined back pain as long-lasting (bothersome pain for more than 1-month) and used a recall period of 12-months. It is uncertain, if such long-lasting pain would be prevalent at such a young age, and if so, if the duration could be clearly identified after up to one year.

Our librarian-assisted search was simple and articles easy to screen. There were also relatively few hits to consider. We therefore do not believe that it was a weakness of this study that only one of the authors screened the initial records for suitable articles. Still, it was of course possible that some relevant articles passed by our search. At the request of one of the reviewers, we did an extra search in PsycINFO and CINAHL, which resulted in no additional material.

The strengths of this review were that it was conducted by two independent persons with no interest in the outcome and that it used a check-list that was specifically designed to suit the purposes of the study. Furthermore, it followed the AMSTAR recommendations for systematic reviews [16]. There was generally good agreement between the reviewers and any queries were easily resolved without the need for a referee. Other reviewers might have preferred to use other descriptive or quality criteria, which could have brought forth other aspects or possibly, other results. A particular strength was that other causal aspects in addition to associations were taken into account in an attempt to establish causality.

## Conclusions

The results of this study show that puberty and back pain are indeed associated and that there are indications towards a causal link.

Nevertheless, causality cannot be established from the studies conducted to date, mainly because large, well-conducted longitudinal studies are lacking.

Further research appears merited, for example focusing on specific hypotheses, such as investigating if there could be a hormonal, biomechanical, behavioral, psychological or nutritional aspect to the development of back pain at this time of life.

In future research, it would be helpful if authors: 1) performed longitudinal studies, 2) used study populations with a suitable mix of children who have and have not started puberty, 3) used relevant definitions of back pain and absence of back pain, 5) took into account the various stages of puberty, 5) showed their estimates, and 6) used statistical tests that suit their data.

## Authors- contributions

All the authors contributed to this review. AL searched in databases. AL and CLY designed the checklists, reviewed the literature, selected and assessed the articles, and analyzed the data. NW helped with the statistical interpretation. AL wrote the first draft. CLY, CLS and NW provided critical comments for the subsequent drafts. All the authors reviewed the final manuscript and approved the final version.

## Authors- information

Arnaud Lardon, DC, MSc, is PhD student at the University of Paris-Sud. Charlotte Leboeuf-Yde, DC, MPH, PhD is Professor in Clinical Biomechanics at the University of Southern Denmark and a Visiting Professor at the University of Paris-Sud with a special interest in the epidemiology of back pain. Christine Le Scanff, PhD, is Professor at the University of Paris-Sud and Director of the Doctoral School 456, with her area of expertise in sports sciences. Niels Wedderkopp, PhD, is Professor in Clinical Biomechanics at the University of Southern Denmark with a special interest in the epidemiology of back pain and sports.

## Additional file

## Electronic supplementary material

Additional file 1: Method for estimation of the odds ratio for back pain in relation to the subsequent pubertal stages using STATA. (DOCX 73 kb) (DOCX 73 KB)

## Authors’ original submitted files for images

Below are the links to the authors’ original submitted files for images. Authors’ original file for figure 1

## Abbreviations

PDS: Pubertal development scale
OR: Odds ratio
CI: Confidence interval
