# Supplementary material for: Is puberty a risk factor for back pain in the young? a systematic critical literature review
Source: Chiropr Man Therap. 2014 Oct 15;22:27. doi: 10.1186/s12998-014-0027-6 (PMC4200222; doi:10.1186/s12998-014-0027-6)
Supplement: Supplementary file 1 — Additional file 1: Method for estimation of the odds ratio for back pain in relation to the subsequent pubertal stages using STATA. (DOCX 73 kb) (DOCX 73 KB) [file 12998_2014_27_MOESM1_ESM.docx]

Appendix 1. Method for estimation of the odds ratio for back pain in relation to the subsequent pubertal stages using STATA.

To calculate the odds ratios (OR) for the various pubertal stages, the OR provided from the logistic regression would serve as the basis for the subsequent values. The log-file in STATA used for this is shown below for the unadjusted OR values of Jansens *et al.* [33] as an example.

name: < OR calc.log >

log: /Users/ OR calc.log

log type: text

opened on: 26 May 2014, 14:55:59

. display log(1.34)

.29266961

. display exp(0*0.29267)

1

. display exp(1*0.29267)

1.3400005

. display exp(2*0.29267)

1.7956014

. display exp(3*0.29267)

2.4061068

. log close

This calculation can also be done by hand, in which case the following procedure is used. One takes the exponential function of the coefficients, thus creating the odds ratios of the separate stages of puberty for back pain.

Stage 1 exp(0)=1 (reference)

Stage 2: exp(0.29267) = 1.34

Stage 3: exp(2*0.29267)=1.79

Stage 4: exp(3*0.29267)=2.4

Both methods would arrive at the same values, namely that the index value (which is set at 1 by definition) is followed by 1.34 at stage 2 (the one obtained in the logistic regression), and 1.79 at stage 3 and 2.4 at stage 4, respectively, calculated as shown above.
