# Supplementary figures and images for: Is puberty a risk factor for back pain in the young? a systematic critical literature review
Source: Chiropr Man Therap. 2014 Oct 15;22:27. doi: 10.1186/s12998-014-0027-6 (PMC4200222; doi:10.1186/s12998-014-0027-6)

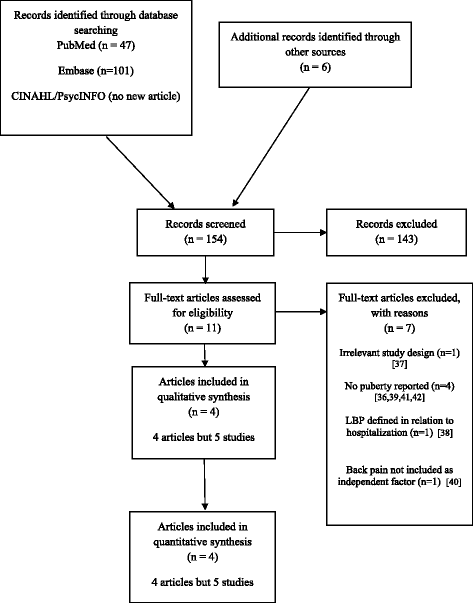

Supplement: Supplementary file 2 — Authors’ original file for figure 1 [file 12998_2014_27_MOESM2_ESM.gif]
